# A Closer Look at Radiation Exposure During Percutaneous Cryoablation for T1 Renal Tumors

**DOI:** 10.3390/cancers17122016

**Published:** 2025-06-17

**Authors:** Luna van den Brink, Michaël M. E. L. Henderickx, Otto M. van Delden, Harrie P. Beerlage, Daniel Martijn de Bruin, Patricia J. Zondervan

**Affiliations:** 1 Department of Urology, Amsterdam UMC, University of Amsterdam, Meibergdreef 9, 1105 AZ Amsterdam, The Netherlands; l.vandenbrink@amsterdamumc.nl (L.v.d.B.);; 2 Cancer Center Amsterdam, Imaging and Biomarkers, 1105 AZ Amsterdam, The Netherlands; 3 Department of Radiology and Nuclear Medicine, Amsterdam UMC, University of Amsterdam, Meibergdreef 9, 1105 AZ Amsterdam, The Netherlands; 4 Department of Biomedical Engineering and Physics, Amsterdam UMC, University of Amsterdam, Meibergdreef 9, 1105 AZ Amsterdam, The Netherlands

**Keywords:** renal cell carcinoma, cryoablation, small renal mass

## Abstract

**Introduction:** Percutaneous cryoablation (PCA) can be a valid alternative to partial nephrectomy for patients with cT1a renal tumors. A potential disadvantage of PCA is radiation exposure for patients, though the exact significance of this is unknown. This study aims to uncover the degree of radiation exposure during PCA and what factors are of influence. **Methods:** This is a retrospective analysis of a prospectively maintained database of patients who underwent CT-guided PCA for cT1 renal cell carcinoma (RCC) between January 2014 and September 2024. The median effective dose (mSV) of PCA was calculated and compared to the expected cumulative radiation exposure during follow-up. Multivariate linear regression was performed to identify factors predictive of higher radiation exposure (mSV). **Results**: A total of 164 PCAs were performed, with radiation data available for 133 cases. Mean age was 65 (±11) years and the mean tumor diameter was 28 (±9.6) mm. Median effective dose of the CA procedures was 26 mSV (IQR 18–37). The estimated cumulative effective dose of follow-up CT scans according to 2016 and 2024 European Association of Urology guidelines was 158 (IQR 117–213) and 105 mSV (IQR 78–142), respectively. Multivariate linear regression analysis identified BMI (OR 1.723, *p* < 0.001), the number of needles used (OR 4.060, *p* < 0.001), and the necessity for additional procedures (OR 8.056, *p* < 0.001) as significant predictors of a higher effective dose. **Conclusions:** We found a median effective dose of 26 mSV for PCA, which is relatively low compared to the cumulative radiation exposure associated with CT scans during follow-up of patients post-ablation according to the guidelines. Furthermore, increased BMI, a higher number of required needles and the execution of additional procedures are all associated with a higher effective dose.

## Simple Summary

Radiation Exposure During Kidney Tumor Cryoablation Percutaneous cryoablation (PCA) is a minimally invasive treatment for renal tumors, offering an alternative to surgical removal. However, this CT-guided procedure exposes patients to radiation, and little is known about the magnitude of this. This study analyzed 164 PCA procedures performed on kidney cancer patients between 2014 and 2024. The radiation exposure of each procedure was measured and was compared to the estimated radiation exposure acquired during follow-up after treatment. The key findings showed that the median radiation dose during PCA was 26 mSv (millisieverts), which is relatively modest. To put this in perspective, patients typically receive much more radiation from the multiple CT scans required for long-term monitoring after treatment - between 105-158 mSv depending on which urological guidelines are followed. Several factors increased radiation exposure during the procedure, such as patients with higher body mass index, procedures requiring more needles, and cases requiring hydro- or aero-dissection all resulted in higher radiation doses. Our study concludes that while PCA does involve radiation exposure, the amount is reasonable compared to the ongoing surveillance scans. The procedure remains a viable treatment option for small kidney tumors, with radiation risk being manageable and predictable based on patient and procedural factors.

## 1. Introduction

The increasing use of cross-sectional imaging has resulted in more frequent detection of small renal masses (SRMs), defined as renal tumors ≤ 4 cm [1]. Although European Association of Urology (EAU) guidelines report partial nephrectomy as the gold standard for treatment of SRMs, alternative kidney sparing treatments such as thermal ablation have gained popularity. Moreover, the guidelines consider CA as a valid treatment option for frail and/or comorbid patients with an SRM [2].

Ablative therapies have evolved rapidly over the past two decades due to the collaboration between urologists, interventional radiologists, biomedical engineers and radiation oncologists [3]. Cryoablation (CA), microwave ablation (MWA), radiofrequency ablation (RFA), stereotactic ablative radiotherapy (SABR) and irreversible electroporation (IRE) have advanced significantly in the past twenty years, with CA emerging as a well-established treatment option for SRM currently. CA has shown favorable oncological outcomes for SRMs, with local recurrence rates ranging from 2 to 8% [4,5]. This technique can be performed using a laparoscopic or percutaneous approach. Both approaches demonstrate success rates exceeding 95% and show no significant differences in complication rate, overall survival, disease-specific survival or recurrence-free survival. Nonetheless, percutaneous cryoablation (PCA) has become more commonly applied in current clinical practice due to the trend towards more minimally invasive techniques [6,7]. Studies show that for patients with tumors ranging from 4 to 7 cm, the risk of disease progression is higher after PCA [8,9]. Nevertheless, it is still employed for patients who are unfit for surgery [10].

In comparison to partial nephrectomy, advantages of PCA include lower risk of complications, a shorter hospital stay and that it is relatively non-invasive [11,12]. In the majority of PCA procedures, computed tomography (CT) imaging is utilized for planning, placement of the cryo-needles, during freezing cycles to monitor the enlarging ice ball and after freezing to assess whether the CA is complete and to establish if there are any per-ablative complications, which makes radiation exposure an inherent aspect of this technique. The literature shows that ionizing radiation can cause DNA damage, and in some cases misrepair can result in point mutations, chromosomal translocations and gene infusions. These changes are associated with an increased risk of malignancies when a cumulative radiation threshold is crossed [13,14,15]. A meta-analysis based on 111.6 million participants showed that the lifetime attribution risk (LAR) for cancer in adults following CT scans was increased (odds ratio [OR] 10) compared to adults exposed only to global background radiation (defined as 2.4 mSv per year). Cancer risk increased with a high radiation dose (OR 33) and multiple CT scan sites (OR 14) [16]. According to the ALARA-principle, physicians should strive to keep radiation exposure ‘As Low As Reasonably Achievable’ to decrease the risk of negative long-term effects of radiation for patients and medical staff whilst maintaining high image quality and providing curative treatment [17].

The literature investigating radiation exposure during PCA for the treatment of renal masses is scarce and the cohorts in these studies are small [18,19]. However, one study reported substantial radiation doses, reaching up to 8267 mG*cm, with authors suggesting that interventional radiologists should be alerted to prompt dose reduction protocols [20]. Given these findings, it is if importance to quantify the radiation exposure in a larger cohort and determine its significance.

This study aims to quantify the radiation exposure associated with PCA and to investigate factors that are associated with higher radiation exposure during this procedure. Additionally, we aim to compare the radiation exposure of PCA to the estimated exposure during follow-up after treatment.

## 2. Methods

### 2.1. Data Collection

This is a retrospective analysis of a prospectively maintained database of patients who underwent PCA in a tertiary referral center between January 2014 and September 2024. Approval to conduct this study was given by our Institutional Review Board (W22_296#22.357). Patients for whom the radiation data were available were extracted for analysis. Radiation data consisted of the dose length product (DLP) in mGy·cm, which was retrieved from the Picture Archiving Communication System (PACS). The DLP was converted into the effective dose (mSV) by using the conversion factor specific to the anatomical region (0.015 mSV·mGy^−1^·cm^−1^ for adult CT abdomen or pelvis scans), which estimates the intra-abdominal radiation based on the output from the scanner [18,21]. An online calculator was used, taking into account the age of the patient and the targeted body part. In order to depict the overall radiation exposure associated with CT scans made during follow-up, an estimation was made of the cumulative radiation exposure, assuming complete follow-up by all patients. The cumulative radiation exposure according to 2016 and 2024 EAU guidelines was estimated using the median effective dose of the first CT chest and abdomen made during follow-up.

### 2.2. PCA Procedure

Patients were discussed in a multidisciplinary meeting to determine the indication for PCA. Final decision on the treatment was made in a shared decision setting between urologist and patient. All patients underwent a renal tumor biopsy. Ideally, this was performed during the diagnostic process prior to the treatment, as described in a study by Widdershoven et al. [22]. Still, due to different factors, in some cases the biopsy was performed in the same session as the PCA.

The percutaneous procedures were performed under CT guidance by an experienced interventional radiologist and assistance was given by a dedicated urologist, as described by Truesdale et al. [23].

Procedures were performed under general anesthesia. After prone or lateral decubitus positioning of the patient, a spiral CT was performed to visualize the renal mass. Then, the cryoprobes were positioned under CT guidance (Siemens Sensation 64 slice, Siemens, Erlangen, Germany). If deemed necessary by the interventional radiologist and urologist, hydro- or aero-dissection was performed to prevent complications. The number and type of cryoprobes (Galil Medical^®^; Arden Hills, MN, USA) was dependent on the volume of the tumor. Short acquisition CT scans were made during the placement of the cryoprobes. The number of scans was dependent on the number of cryoprobes and whether adjustment of the probes was necessary. The freeze–thaw cycle was performed twice and consisted of ten minutes of freezing, followed by four minutes of passive thawing and one minute of active thawing. In the earliest PCA, Argon and helium gas were used for freezing and active thawing cycles, respectively, until the helium-free CX cryoprobes (Galil Medical^®^; Arden Hills, MN, USA) were used, making helium gas obsolete. After the last passive thaw cycle, the active thaw cycle was continued until the cryoprobes could easily be removed. During the freezing cycles, several spiral CT scans were made to visualize the ice ball. The goal of the ablation was to create a circumferential ablative margin around the tumor. A control CT scan was performed, during the last freeze cycle, to visualize the ablation area and assess the immediate treatment result and per-procedural complications. In general, patients were hospitalized for one night after the ablation for observation.

### 2.3. Follow-Up

Follow-up imaging was performed at three months to determine persistence by means of a multi-phase CT scan of the abdomen alone or chest and abdomen combined. These follow-up scans consisted of a non-enhanced scan and an arterial and venous scan. According to our institution’s protocol, follow-up scans did not include a CT of the chest, but in some cases, a scan of the chest was also made at the discretion of the treating urologist. If radiological enhancement of the ablation zone was seen at three months follow-up, it was considered persistence [24]. Subsequent follow-up was performed according to EAU guideline recommendations. Notably, EAU guideline recommendations regarding radiological follow-up after ablative therapy have changed in the past decade. Up to 2017, patients who underwent CA were classified as intermediate/high risk of recurrence and underwent more frequent imaging during follow-up compared to current guideline recommendations. The 2024 EAU guidelines do not distinguish CA from surgical treatment for follow-up imaging, thus presumably most focal ablation cases are considered low risk based on tumor size and histology from renal tumor biopsies. [2]. Table 1 shows follow-up schedules post-ablation according to different guidelines.

### 2.4. Statistical Analysis

Descriptive statistics were performed to show baseline characteristics and were expressed as means, medians or percentages. The effective dose (mSV) was expressed as median with interquartile range (IQR). The cumulative radiation exposure during follow-up was estimated according to the 2016 and 2024 EAU guidelines including the first CT scan at 3 months to determine persistence. Assuming complete follow-up, this corresponds to nine CT scans according to 2016 guidelines and six according to 2024 guidelines. Multivariate linear regression was performed to identify factors that are predictive of a higher effective dose during PCA procedures. The backward elimination method was used for multivariate regression. Statistical analyses were performed using SPSS version 28 (IBM Corp., Armonk, NY, USA).

## 3. Results

### 3.1. Baseline Characteristics

A total of 164 PCA procedures were performed of which radiation data was available for 133 cases. The mean age of the included patients was 65 years (±11) and the average diameter of the lesion was 28 mm (±9.6). RENAL nephrometry scores were distributed as follows: 56 patients (42%) had low anatomical complexity, 70 patients (53%) had intermediate, and 7 patients (5.3%) had high anatomical complexity. The median number of CT scans performed per procedure was 16 (11–36). Baseline characteristics are shown in Table 2. In 45 (34%) patients, procedures such as hydro- or aero-dissection were conducted during PCA. There were 13 cases of hydro-dissection, 18 cases of aero-dissection and 11 patients had both. In three cases an artificial pneumothorax was performed. More details on additional procedures are displayed in Supplementary Table S1. Three patients suffered from complications higher than Clavien Dindo grade 3. Two patients suffered a cerebral infarction the day after the PCA procedure requiring intervention, and one patient experienced neuropathic pain due to nerve damage requiring intervention by means of a nerve block.

### 3.2. Effective Dose

The median effective dose of a PCA procedure was 26 mSV (IQR 18–37). Follow-up imaging by means of a multiphase CT scan, of the abdomen alone or chest and abdomen combined, was available in 116 patients. There were 13 patients who had MRI and/or ultrasound during follow-up instead of CT scans. Follow-up information was not available for four patients as they were referred back to their referring urologist for follow-up.

**Figure 1:**
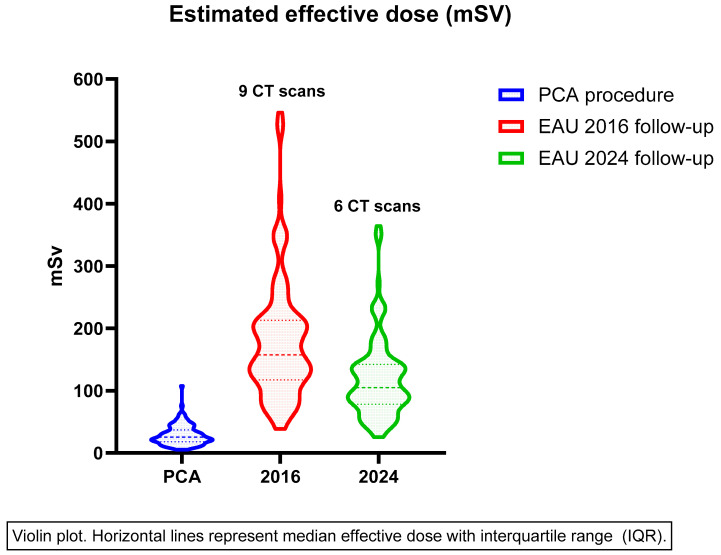
Effective dose of PCA procedure and estimated cumulative effective dose of CT scans during follow-up.

The estimated cumulative effective dose of follow-up CT scans according to 2016 and 2024 EAU guidelines was 10 mSV (IQR 78–142) and 154 (IQR 117–213), respectively. The median effective doses of PCA and follow-up imaging are displayed in the violin plot in Figure 1. The median effective dose of PCA fluctuated slightly over the years, though it appears to remain stable between 2019 and 2024 as shown in Supplementary Figure S1.

### 3.3. Factors Influencing Radiation Exposure

Multivariate linear regression showed BMI (OR 1.727, *p* < 0.001), increasing the number of needles (OR 4.323, *p* < 0.001) and additional procedures (OR 7.632, *p* = 0.002) to be predictive for the effective dose, though the regression coefficient was low (R^2^ = 0.410, *p* < 0.001). Regression analyses are shown in Table 3. Age and RENAL score were not associated with increased radiation exposure.

## 4. Discussion

To date, CA is the most studied ablative technique and is a kidney-sparing alternative offered to patients with an SRM who are unable to undergo partial nephrectomy. This study quantified the radiation exposure during PCA and investigated factors contributing to increased radiation exposure during this procedure. We found a median effective dose of 26 mSV for a PCA procedure. Factors such as a higher BMI, a greater number of needles and the performance of hydro- or aero-dissection during PCA were correlated with a higher effective dose, whereas RENAL score and age were not.

Although the literature on this topic is limited, some small retrospective cohort studies have described radiation exposure during PCA prior to this study. A retrospective study by Tracy et al. also reported the radiation exposure for patients who underwent PCA and found a median effective dose of 30 mSV (IQR 21–47), which is similar to the findings in this study [18]. On the other hand, a study by Arnold et al. evaluated radiation exposure in a cohort of 30 patients and found a higher effective dose for PCA procedures, with an average of 40 mSV [25]. Finally, Bjorgberg et al. found a lower effective dose for PCA procedures (18 mSV), despite using the same CT scanner and number of cryoprobes as in our center, as well as a patient group with a comparable BMI [26]. This discrepancy may be attributable to the higher number of CT scans made during different phases of the PCA procedure at our institution. The amount of CT scans made during PCA procedures can vary among interventional radiologists due to preference or experience. While monitoring with spiral CT allows for the early detection of peri-operative complications and optimal evaluation of the ice ball, thereby maximizing the chance of freezing the entire lesion and minimizing the risk of damage to surrounding organs, these CT scans contribute to the overall radiation exposure [27].

In this study, factors such as BMI, the amount of needles used and the performance of hydro- and aero-dissection were associated with an increased effective dose. Obese patients often require a higher effective dose to minimize scattered radiation and maintain image quality [28]. A retrospective study by Fukushima et al. found that increased radiation exposure, expressed as the entrance skin dose (ESD), was associated with a higher number of cryoprobes and the combined use of hydro- and aero-dissection [18]. It can be expected that performing hydro- or aero-dissection will require more spiral CT scans, thereby causing the effective dose to increase. The same applies for the use of more cryoprobes, as the positioning of each probe is verified through intermittent CT scans. Also, cryoprobes are highly attenuating, and can cause artifacts in case the radiation dose is too low [29]. Lastly, factors such as the length of the scan and the desired image quality, which is subjective per physician, can also contribute to the effective dose [30]. As PCA gains a more prominent role in the treatment of SRMs, interventional radiologists should collaborate to identify where radiation exposure can be reduced when possible. Nonetheless, when physicians determine an indication for a CT scan, the benefit of radiation exposure should exceed the harm of an increased risk of malignancies [12].

Arguably, the significance of the radiation burden associated with the PCA procedure is questionable. In fact, it appears to be relatively minor when compared to the cumulative radiation exposure from CT scans during follow-up after ablative therapy. The BEIR VII model, based on studies of survivors of the atomic bombings in Hiroshima and Nagasaki, and on studies regarding occupational and medical exposures to radiation, estimates the risk of radiation-induced malignancies associated with CT scans. The American College of Radiology created a calculator based on this model to determine the LAR for developing cancer, incorporating patient age, gender and the type of exam [31]. For example, a 65-year old male has a lifetime cancer risk of 45%, and an additional risk of 0.09% after undergoing PCA for treatment of an SRM. Assuming follow-up according to the 2024 and 2016 EAU guidelines, there would be an additional cancer risk of 0.35% and 0.53%, respectively. Therefore, the additional cancer risk associated with PCA seems low when compared to radiological follow-up schedules proposed by the guidelines. Follow-up schedules proposed by international guidelines lack evidence, thus research should be carried out to determine if a reduction in CT scans is warranted in order to decrease radiation exposure. Levesque et al. described that decreasing the number of monitoring scans and a reduction in the current and kilovoltage of targeting and monitoring scans can reduce the radiation dose. Furthermore, the use of CT fluoroscopy instead of helical CT’s during targeting and monitoring can lead to further reduction [20].

Nonetheless, procedures such as ultrasound-guided or cone beam CT PCA serve as alternative ablation techniques that offer a lower radiation dose, with a mean effective dose of 4.3 mSv for abdominal cone beam CT [32]. A case series with ultrasound-guided targeting and CT-guided ice ball monitoring PCA showed promising short-term results with technical success achieved in 100% and a 1-year progression-free survival rate of 96% [33]. A recently published study comparing cone beam CT-guided PCA with CT-guided PCA found a recurrence-free survival (RFS) of 90% at a median follow-up of 40 months for cone beam and 88% for CT-guided PCA (*p* = 0.083) [34]. However, other series report a higher RFS for CT-guided ablation in larger cohorts [34,35,36]. Compared to CA, CT-guided RFA has shown a lower radiation exposure [37]. This can be explained by factors that are inherent to each specific treatment. RFA generally requires only one needle, whereas cryoablation requires multiple. The placement of these needles will then again require more imaging to assure correct positioning. However, even though CA has a higher radiation dose, it does have a great advantage in regard to RFA or other ablative techniques. Namely, one can visualize the extent of the ice-ball during the procedure. This allows the treating physician to treat the entire tumor with a lower change in persistence and a lower risk of complications (i.e., damage to adjacent structures) [20].

This study is one of the few studies uncovering the radiation exposure associated with PCA and reported a relatively large series of patients of a prospectively maintained database. There are some limitations to this study. First, even though this study is based on a prospectively maintained database including all procedures, the major limitation of these results lies within the retrospective nature of the study design. Second, this is a single-center study, thus it may not be reflective of patient populations in other institutions. Nonetheless, the effective dose found in this study did not differ greatly from priorly reported radiation doses in other institutions. Third, the data in this study comprises a timeframe of 10 years, meaning there could be a learning curve in performing PCAs, potentially influencing the number of CT scans made during the procedure. However, despite some fluctuation in the first five years, we did not see a significant difference in the effective dose from 2014 until 2024. Lastly, radiation data was missing in 31 patients, all of whom were treated in the beginning of the study period, which could have impacted the median effective dose.

## 5. Conclusions

This study found a median effective dose of 26 mSV for a PCA procedure. Though factors such as a higher BMI, the use of more cryoprobes and the performance of hydro- or aero-dissection seem to correlate with a higher effective dose, the overall radiation exposure of PCA is relatively low, specifically when compared to the estimated cumulative effective dose of surveillance CT scans.

## Figures and Tables

**Table 1:** Guideline recommendations for follow-up after thermal ablation.

Months Post-Ablative Therapy										
	3	6	12	18	24	30	36	48	60	>60
EAU 2024		CT		CT		CT		>3 yrs CT once every 2 yrs		
EAU 2016		CT	CT		CT		CT	CT	CT	CT once every 2 yrs up to 10 yrs
AUA 2021		CT	CT		CT		CT	CT	CT	Every 2 yrs up to 10 yrs
NCCN 2024	CT/MRI/CEUS	CT/MRI/CEUS	CT/MRI/CEUS		CT/MRI/CEUS		CT/MRI/CEUS	CT/MRI/CEUS	CT/MRI/CEUS	

Abbreviations: European Association of Urology (EAU), American Urological Association (AUA), National Comprehensive Cancer Network (NCCN), computed tomography (CT), magnetic resonance imaging (MRI), contrast-enhanced ultrasound (CEUS), years (yrs).

**Table 2:** Baseline characteristics of patients who underwent PCA.

Variable	N = 133
Age (mean, stdev)	65 (11)
Gender, male (%)	93 (70)
Side, left (%)	63 (47)
BMI (mean, stdev)	29 (5.2)
Serum creatinine (med IQR)	95 (77–116)
eGFR (median, IQR)	70 (50–83)
Tumor diameter, mm (mean, sdtev)	28 (9.6)
RENAL group (%)	
Low (4–6)	56 (42)
Intermediate (7–9)	70 (53)
High (10–12)	7 (5.3)
No. of needles (mean, stdev)	3.6 (1.0)
Additional procedures	45 (34)
Complications Clavien Dindo ≥3	3 (2.3)

**Table 3:** Multivariate analysis of factors associated with a higher effective dose.

Variable	OR	95% CI	*p*-Value
BMI	1.723	1.315, 2.132	<0.001
Number of needles	4.060	2.019, 6.102	<0.001
Additional procedures	8.056	2.019, 6.102	<0.001

## Data Availability

Data can be shared upon request.

## Supplementary Materials

The following supporting information can be downloaded at https://www.mdpi.com/article/10.3390/cancers17122016/s1: Supplementary Table S1: Additional procedures performed during PCA procedure. Supplementary Figure S1: Median effective dose of PCA from 2014 to 2024.

## References

[1] Capitanio U., Bensalah K., Bex A., Boorjian S.A., Bray F., Coleman J., Gore J.L., Sun M., Wood C., Russo P. (2019). Epidemiology of Renal Cell Carcinoma. Eur. Urol..

[2] Ljungberg B., Albiges L., Abu-Ghanem Y., Bensalah K., Dabestani S., Fernández-Pello S., Giles R.H., Hofmann F., Hora M., Kuczyk M.A. (2022). European Association of Urology Guidelines on Renal Cell Carcinoma: The 2022 Update. Eur. Urol..

[3] Zondervan P.J., Buijs M., De Bruin D.M., van Delden O.M., Van Lienden K.P. (2019). Available ablation energies to treat cT1 renal cell cancer: Emerging technologies. World J. Urol..

[4] Nielsen T.K., Lagerveld B.W., Keeley F., Lughezzani G., Sriprasad S., Barber N.J., Hansen L.U., Buffi N.M., Guazzoni G., van der Zee J.A. (2017). Oncological outcomes and complication rates after laparoscopic-assisted cryoablation: A European Registry for Renal Cryoablation (EuRECA) multi-institutional study. BJU Int..

[5] Pickersgill N.A., Vetter J.M., Kim E.H., Cope S.J., Du K., Venkatesh R., Giardina J.D., Saad N.E., Bhayani S.B., Figenshau R.S. (2020). Ten-Year Experience with Percutaneous Cryoablation of Renal Tumors: Tumor Size Predicts Disease Progression. J. Endourol..

[6] Pessoa R.R., Autorino R., Laguna M.P., Molina W.R., Gustafson D., Nogueira L., da Silva R.D., Werahera P.N., Kim F.J. (2017). Laparoscopic Versus Percutaneous Cryoablation of Small Renal Mass: Systematic Review and Cumulative Analysis of Comparative Studies. Clin. Genitourin. Cancer.

[7] Goyal J., Verma P., Sidana A., Georgiades C.S., Rodriguez R. (2012). Single-center comparative oncologic outcomes of surgical and percutaneous cryoablation for treatment of renal tumors. J. Endourol..

[8] Hebbadj S., Cazzato R.L., Garnon J., Shaygi B., Buy X., Tsoumakidou G., Lang H., Gangi A. (2018). Safety Considerations and Local Tumor Control Following Percutaneous Image-Guided Cryoablation of T1b Renal Tumors. Cardiovasc. Intervent. Radiol..

[9] Grange R., Tradi F., Izaaryene J., Daidj N., Brunelle S., Walz J., Gravis G., Piana G. (2019). Computed tomography-guided percutaneous cryoablation of T1b renal tumors: Safety, functional and oncological outcomes. Int. J. Hyperth..

[10] Pecoraro A., Palumbo C., Knipper S., Mistretta F.A., Tian Z., Shariat S.F., Saad F., Briganti A., Fiori C., Porpiglia F. (2019). Cryoablation Predisposes to Higher Cancer Specific Mortality Relative to Partial Nephrectomy in Patients with Nonmetastatic pT1b Kidney Cancer. J. Urol..

[11] Zondervan P.J., Buijs M., de la Rosette J.J., van Delden O., van Lienden K., Laguna M.P. (2016). Cryoablation of small kidney tumors. Int. J. Surg..

[12] Garnon J., Van Strijen M.J., Nielsen T.K., King A.J., Montauban Van Swijndregt A.D., Cazzato R.L., Auloge P., Rousseau C., Dalili D., Keeley F.X. (2019). Safety of percutaneous renal cryoablation: An international multicentre experience from the EuRECA retrospective percutaneous database. Eur. Radiol..

[13] Linet M.S., Slovis T.L., Miller D.L., Kleinerman R., Lee C., Rajaraman P., Berrington de Gonzalez A. (2012). Cancer risks associated with external radiation from diagnostic imaging procedures. CA Cancer J. Clin..

[14] Koenig T.R., Wolff D., Mettler F.A., Wagner L.K. (2001). Skin injuries from fluoroscopically guided procedures: Part 1, characteristics of radiation injury. AJR Am. J. Roentgenol..

[15] Brenner David J., Hall Eric J. (2007). Computed Tomography—An Increasing Source of Radiation Exposure. New Engl. J. Med..

[16] Cao C.F., Ma K.L., Shan H., Liu T.F., Zhao S.Q., Wan Y., Wang H.Q. (2022). CT Scans and Cancer Risks: A Systematic Review and Dose-response Meta-analysis. BMC Cancer.

[17] Henderickx M.M., Brits T., Zabegalina N.S., Baard J., Ballout M., Beerlage H.P., De Wachter S., Kamphuis G.M. (2022). Can operator-controlled imaging reduce fluoroscopy time during flexible ureterorenoscopy?. Cent. Eur. J. Urol..

[18] Tracy C.R., Kogan P., Gupta A., Gahan J.C., Theckumparampil N.P., Elsamra S.E., Okunov Z., Sun S., Lall C., Lobko I. (2015). Radiation Exposure During Percutaneous Ablation of Small Renal Masses: A Multi-Institutional Multimodality Analysis. J. Endourol..

[19] Fukushima Y., Nakamura J., Seki Y., Ando M., Miyazaki M., Tsushima Y. (2021). Patients’ radiation dose in computed tomography-fluoroscopy-guided percutaneous cryoablation for small renal tumors. Eur. J. Radiol..

[20] Levesque V.M., Shyn P.B., Tuncali K., Tatli S., Nawfel R.D., Olubiyi O., Silverman S.G. (2015). Radiation dose during CT-guided percutaneous cryoablation of renal tumors: Effect of a dose reduction protocol. Eur. J. Radiol..

[21] Tsalafoutas I.A., Koukourakis G.V. (2010). Patient dose considerations in computed tomography examinations. World J. Radiol..

[22] Widdershoven C.V., Aarts B.M., Zondervan P.J., Henderickx M.M., Klompenhouwer E.G., van Delden O.M., Prevoo W., Montauban van Swijndregt A.D., van Moorselaar R.J., Bex A. (2021). Renal biopsies performed before versus during ablation of T1 renal tumors: Implications for prevention of overtreatment and follow-up. Abdom. Radiol..

[23] Truesdale C.M., Soulen M.C., Clark T.W., Mondschein J.I., Wehrenberg-Klee E., Malkowicz S.B., Wein A.J., Guzzo T.J., Stavropoulos S.W. (2013). Percutaneous Computed Tomography–guided Renal Mass Radiofrequency Ablation versus Cryoablation: Doses of Sedation Medication Used. J. Vasc. Interv. Radiol..

[24] Zondervan P.J., Wagstaff P.G.K., Desai M.M., de Bruin D.M., Fraga A.F., Hadaschik B.A., Köllermann J., Liehr U.B., Pahernik S.A., Schlemmer H.P. (2016). Follow-up after focal therapy in renal masses: An international multidisciplinary Delphi consensus project. World J. Urol..

[25] Arnold D.C., Schroeder G., Smith J.C., Wahjudi I.N., Heldt J.P., Richards G.D., Agarwal G., Brisbane W.G., Farley D.V., Baldwin D.D. (2013). Comparing Radiation Exposure Between Ablative Therapies for Small Renal Masses. J. Endourol..

[26] Borgbjerg J., Bylling T., Andersen G., Thygesen J., Mikkelsen A., Nielsen T.K. (2020). CT-guided cryoablation of renal cancer: Radiation burden and the associated risk of secondary cancer from procedural- and follow-up imaging. Abdom. Radiol..

[27] Park B.K., Morrison P.R., Tatli S., Govindarajulu U., Tuncali K., Judy P., Shyn P.B., Silverman S.G. (2012). Estimated effective dose of CT-guided percutaneous cryoablation of liver tumors. Eur. J. Radiol..

[28] Aljweber H.A., Mamoun E., Khouqeer G.A., Elgarayhi A., Sallah M. (2024). Reducing effective radiation dose with improved image quality of abdominal computed tomography scans for overweight patients. J. Radiat. Res. Appl. Sci..

[29] Leng S., Atwell T.D., Yu L., Mandrekar J., Lewis B.D., Woodrum D.A., McCollough C.H. (2011). Radiation Dose Reduction for CT-Guided Renal Tumor Cryoablation. Am. J. Roentgenol..

[30] Zhong J., Gallagher M., Hounslow C., Iball G., Wah T. (2021). Radiation dose reduction in CT-guided cryoablation of renal tumors. Diagn. Interv. Radiol..

[31] X-Ray Risk. https://www.xrayrisk.com/calculator/calculator-normal-studies.php.

[32] Sailer A.M., Schurink G.W.H., Wildberger J.E., de Graaf R., van Zwam W.H., de Haan M.W., Kemerink G.J., Jeukens C.R. (2015). Radiation exposure of abdominal cone beam computed tomography. Cardiovasc. Intervent. Radiol..

[33] Kim D.K., Won J.Y., Park S.Y. (2019). Percutaneous cryoablation for renal cell carcinoma using ultrasound-guided targeting and computed tomography-guided ice-ball monitoring: Radiation dose and short-term outcomes. Acta Radiol..

[34] Duijn M., Ruiter A.E.C., van Swijndregt A.D.M., van der Hulst V.P.M., Lagerveld B.W. (2025). Preliminary Assessment of Cone Beam CT Guided Percutaneous Cryoablation for CT1A Renal Cell Carcinoma: A Relatively Novel and Underutilized Technique. Clin. Genitourin. Cancer.

[35] Duijn M., Ruiter A.E.C., Montauban van Swijndregt A.D., Lagerveld B.W. (2023). P174—The efficacy and safety of cone beam computed tomography for percutaneous renal cell carcinoma cryoablation: A single-center long-term follow-up study. Eur. Urol. Open Sci..

[36] Breen D.J., King A.J., Patel N., Lockyer R., Hayes M. (2018). Image-guided Cryoablation for Sporadic Renal Cell Carcinoma: Three- and 5-year Outcomes in 220 Patients with Biopsy-proven Renal Cell Carcinoma. Radiology.

[37] McEachen J.C., Leng S., Atwell T.D., Tollefson M.K., Friese J.L., Wang Z., Murad M.H., Schmit G.D. (2016). Percutaneous Renal Tumor Ablation: Radiation Exposure During Cryoablation and Radiofrequency Ablation. Cardiovasc. Intervent. Radiol..

