# Supplementary material for: A Closer Look at Radiation Exposure During Percutaneous Cryoablation for T1 Renal Tumors
Source: Cancers (Basel). 2025 Jun 17;17(12):2016. doi: 10.3390/cancers17122016 (PMC12191335; doi:10.3390/cancers17122016)

Supplementary Table S1: Additional procedures performed during PCA procedure

Procedure	Number of cases n=133 (%)
Hydrodissection	13 (9.8)
Pneumothorax	3 (2.2)
Aerodissection	18 (14)
Hydro- and aerodissection	11 (8.3)
None	86 (65)

Supplementary Figure S1: Median effective dose of PCA from 2014 to 2024

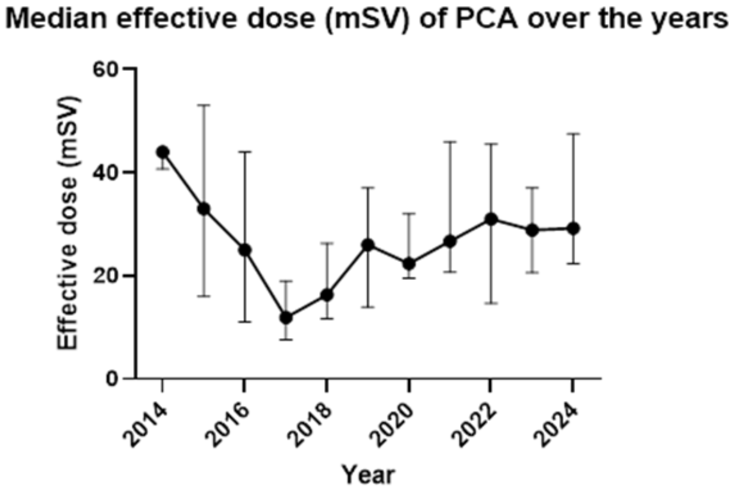

Supplement: Supplementary file 1 [file cancers-17-02016-s001.zip › cancers-3655912-supplementary.pdf]
